# Performance of polygenic risk scores for cancer prediction in a racially diverse academic biobank

**DOI:** 10.1016/j.gim.2021.10.015

**Published:** 2021-11-30

**Authors:** Louise Wang, Heena Desai, Shefali S. Verma, Anh Le, Ryan Hausler, Anurag Verma, Renae Judy, Abigail Doucette, Peter E. Gabriel, Regeneron Genetics Center, Katherine L. Nathanson, Scott M. Damrauer, Danielle L. Mowery, Marylyn D. Ritchie, Rachel L. Kember, Kara N. Maxwell

**Affiliations:** 1 Division of Gastroenterology, Department of Medicine, Perelman School of Medicine, University of Pennsylvania, Philadelphia, PA; 2 Division of Hematology/Oncology, Department of Medicine, Perelman School of Medicine, University of Pennsylvania, Philadelphia, PA; 3 Department of Genetics, Perelman School of Medicine, University of Pennsylvania, Philadelphia, PA; 4 Department of Surgery, Perelman School of Medicine, University of Pennsylvania, Philadelphia, PA; 5 Abramson Cancer Center, University of Pennsylvania, Philadelphia, PA; 6 Department of Radiation Oncology, Perelman School of Medicine, University of Pennsylvania, Philadelphia, PA; 7 Division of Translational Medicine and Human Genetics, Department of Medicine, Perelman School of Medicine, University of Pennsylvania, Philadelphia, PA; 8 Corporal Michael J. Crescenz VA Medical Center, U.S. Department of Veterans Affairs, Philadelphia, PA; 9 Department of Biostatistics, Epidemiology, and Informatics, Perelman School of Medicine, University of Pennsylvania, Philadelphia, PA; 10 Department of Psychiatry, Perelman School of Medicine, University of Pennsylvania, Philadelphia, PA

**Keywords:** Cancer risk, GWAS, Polygenic risk score, PRS, Racial differences

## Abstract

**Purpose:**

Genome-wide association studies have identified hundreds of single nucleotide variations (formerly single nucleotide polymorphisms) associated with several cancers, but the predictive ability of polygenic risk scores (PRSs) is unclear, especially among non-Whites.

**Methods:**

PRSs were derived from genome-wide significant single-nucleotide variations for 15 cancers in 20,079 individuals in an academic biobank. We evaluated the improvement in discriminatory accuracy by including cancer-specific PRS in patients of genetically-determined African and European ancestry.

**Results:**

Among the individuals of European genetic ancestry, PRSs for breast, colon, melanoma, and prostate were significantly associated with their respective cancers. Among the individuals of African genetic ancestry, PRSs for breast, colon, prostate, and thyroid were significantly associated with their respective cancers. The area under the curve of the model consisting of age, sex, and principal components was 0.621 to 0.710, and it increased by 1% to 4% with the inclusion of PRS in individuals of European genetic ancestry. In individuals of African genetic ancestry, area under the curve was overall higher in the model without the PRS (0.723-0.810) but increased by <1% with the inclusion of PRS for most cancers.

**Conclusion:**

PRS moderately increased the ability to discriminate the cancer status in individuals of European but not African ancestry. Further large-scale studies are needed to identify ancestry-specific genetic factors in non-White populations to incorporate PRS into cancer risk assessment.

## Introduction

Cancer is the second leading cause of death nationally, with >1.8 million new cancer cases and 600,000 deaths projected in the United States in 2020.^1^ The development of risk-stratification models on the basis of a combination of genetic and nongenetic risk factors allows development of personalized cancer prevention and surveillance strategies to reduce cancer morbidity and mortality.^1–3^ For example, identification of rare genetic factors of high and moderate risk, such as *BRCA1/2* pathogenic variants’ status, is used to determine cancer surveillance and prevention strategies for breast cancer.^4,5^ However, these factors account for <5% of breast cancer prevalence in large, population based studies.^6,7^ Current strategies for breast cancer risk prediction for most women rely upon models that incorporate nongenetic breast cancer risk factors such as age, race, family history,^8^ estrogen-related factors,^9^ body mass index (BMI), and mammographic density.^10^

Incorporation of common genetic factors could vastly improve the current risk prediction models for cancer. Genome-wide association studies (GWAS) have identified a large number of common genetic variants associated with multiple cancers,^11^ but the risk association of each variant is small and impossible to individually incorporate into risk prediction models. Polygenic risk scores (PRSs) are a weighted sum of multiple disease associated alleles to identify individuals at high risk for a specific disease or phenotype.^12^ For example, PRS can stratify women above and below a lifetime breast cancer risk threshold of 20% that is used to justify the incorporation of breast magnetic resonance imaging into cancer surveillance protocols.^13^

PRS improves the accuracy of breast cancer risk prediction models;^2,4^ however, PRS is less studied in other cancer types.^12^ In addition, because non-European patients are historically underrepresented in GWAS,^14^ the weights derived from European GWAS and used for PRS models may not be appropriate for non-White patients.^15^ Current commercial genetic tests using PRS are only available for individuals of European and/or Ashkenazi Jewish ancestry, which may worsen already existing cancer health disparities.^16^

We therefore sought to evaluate the improvement in discriminatory ability with the inclusion of PRS in predicting cancer risk in genetically determined African and European individuals from an academic biobank for 15 cancers (bladder, breast, colorectal, endometrial, esophageal, glioma, lung, melanoma, oral cavity and pharynx, ovarian, pancreatic, prostate, renal, testicular, and thyroid) that had at least 1 GWAS within the past 10 years.

## Materials and Methods

### Penn Medicine BioBank cohort and genotyping

The Penn Medicine BioBank (PMBB) is a genomic and precision medicine cohort comprising participants who actively consent for biospecimen collection and linkage of their biospecimen to their electronic health record (EHR) data.^17^ Participants were recruited into PMBB between 2004 and 2020 to a University of Pennsylvania institutional review board approved study at the time of medical appointments in the University of Pennsylvania Health System (N = 60,232). Genotyping was ongoing as samples were recruited. As of December 2020, 20,079 unique participants (some samples were genotyped multiple times) had genome-wide DNA array-based genotyping using the Infinium Global Screening Array (GSA) chip (Illumina) in 3 batches: (1) 5676 samples on Illumina GSA V1 chip (single-nucleotide variations [SNVs] = 700,078 [formerly single-nucleotide polymorphisms]), (2) 2972 samples on Illumina GSA V2 chip at Children’s Hospital of Philadelphia Center for Applied Genomics (SNVs = 759,993), and (3) 16,940 samples on Illumina GSA V2 chip at Regeneron Genetics Center. All individuals who were recruited for the PMBB were patients of clinical practice sites of the University of Pennsylvania Health System. Appropriate consent was obtained from each participant regarding storage of biological specimens, genetic sequencing, access to all available EHR data, and permission to recontact for future studies. The study was approved by the Institutional Review Board of the University of Pennsylvania.

### Genotyping quality control and genetic ancestry determination

Quality control of the data set consisted of removing individuals with sex mismatch errors (eg, reported sex different from inferred sex) or had a sample call rate of <90% and removing palindromic variants or variants with a call rate of <95%. Genotyping data of unique samples were phased (using EAGLE v2.4.1 software) and imputed to the Trans-Omics for Precision Medicine reference panel (Freeze 5) on the Trans-Omics for Precision Medicine imputation server. Eigenstrat principal components (PCs) analysis was used to identify the genetic substructure of the entire PMBB population, and quantitative discriminant analysis was performed on all samples to determine their genetically informed ancestry. For these analyses, 1000 Genomes samples were used as training set with labels and PMBB samples were used as a testing set to determine their ancestry. Individuals from genetically-determined African and European ancestry were included in the analysis. The first 10 ancestry-specific PCs for the African and European individuals were used as covariates to account for genetic ancestry.^18^

### Phenotyping of PMBB participants

We evaluated 19,935 individuals in PMBB with available genotyping data who had passed genotyping quality control and had at least 1 physician encounter, who together had 4.9 million health care encounters. Cancer cases were identified from the EHR using International Classification of Diseases (ICD)-9 or ICD-10 billing codes. To determine the number of instances of ICD-9/10 codes needed to identify cancer cases, manual chart review was performed for 2365 individuals with at least 1 ICD-9/10 billing code for prostate cancer. The positive predictive value of 1 ICD-9/10 prostate cancer billing code was 94% (Supplemental Figure 1). On the basis of this, we used a similar strategy to identify individuals with bladder, breast, colorectal, endometrial, esophageal, glioma, lung, melanoma, oral cavity and pharynx, ovarian, pancreatic, prostate, renal, testicular, and thyroid cancers (Supplemental Table 1, Supplemental Figure 2). Cases comprised both prevalent and incident cases. In total, there were 18 to 457 and 6 to 421 cancer cases in individuals of European and African genetic ancestry, respectively (Table 1). Controls were defined as individuals with no ICD-9/10 codes for invasive cancer, benign, in situ, or secondary neoplasms (Supplemental Figure 2). The African genetic ancestry data set contained 8711 individuals and the European genetic ancestry data set contained 9788 individuals. Of these, 8673 individuals of African genetic ancestry and 9759 individuals of European genetic ancestry had complete genotype, phenotype, and covariate data.

### SNV selection

Summary statistics were obtained using the most recent study and largest GWAS available in the GWAS catalog^19^ for each of the 15 cancers in this study (Supplemental Table 2). All of the available GWAS were conducted on European ancestry individuals. SNVs were chosen on the basis of a *P* value threshold of *P* < 1 × 10^−6^. SNVs were pruned for linkage disequilibrium (LD) on the basis of pairwise genotypic correlation at *r^2^* = 0.1 (Supplemental Table 3) using the European 1000 Genome database reference panel in PLINK 1.9.^20^ In addition, we also evaluated the performance of previously established PRS for breast (Breast-313)^21^ and prostate cancer^22^ in our biobank. For the previously validated prostate cancer PRS, we calculated PRS using weights from individuals of European (Prostate-EUR) and African (Prostate-AFR) genetic ancestry separately.

### PRS generation and statistical analysis

PRS for each individual was calculated using PLINK 1.9 by summing the LD-pruned SNV variants and weighing their corresponding effect sizes using the odds ratios (ORs) or beta values reported in the original GWAS. We standardized each cancer PRS to a mean of 0 and SD of 1 and used logistic regression to test for the association between cancer PRS and cancer phenotype and to compare the top versus bottom quintiles of polygenic risk, controlling for age, sex, and the first 10 within-ancestry PCs as covariates. We performed a primary analysis for cancers with >100 cases and included a secondary analysis of the remaining cancer types. Area under the curve (AUC) for primary phenotype was determined using the package pROC.^23^ All statistical analyses were performed using R 4.0.3.

## Results

Of the 9759 individuals of European genetic ancestry (men = 6379; women = 3380; mean age = 64.1 years) with complete information (genotype, phenotype, and covariate), 1735 (17.8%) had at least 1 ICD-9/10 code for at least 1 of the 15 different types of cancer (bladder, breast, colorectal, endometrial, esophageal, glioma, lung, melanoma, oral cavity, ovarian, pancreatic, prostate, renal, testicular, thyroid) (Table 1). There were 8673 individuals of African genetic ancestry with complete information (men = 3200; women = 5473; mean age = 51.9 years) in PMBB, and of these, 1420 (16.4%) had at least 1 ICD-9/10 code recorded for a cancer of interest (Table 1).

**Figure 1:**
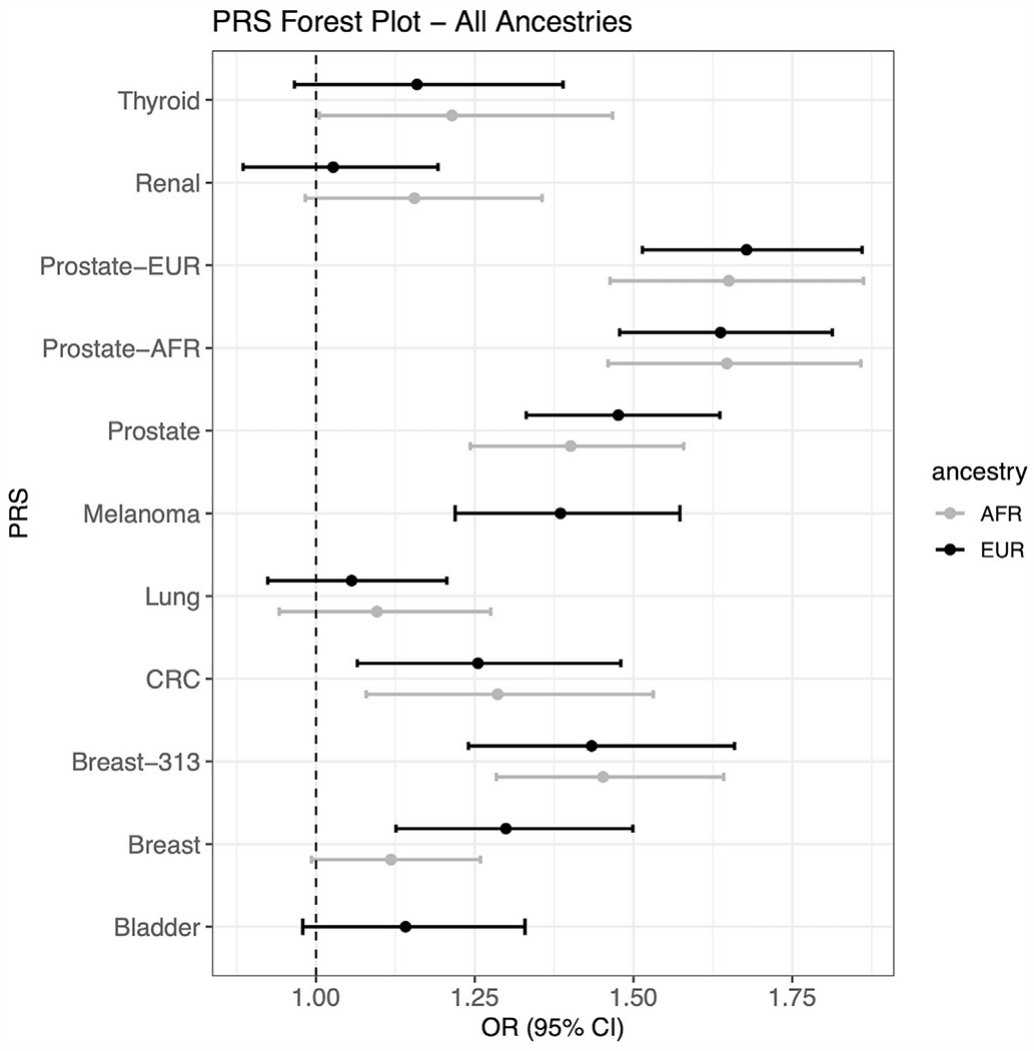
Association of PRS with cancer in individuals of genetically determined African ancestry and European ancestry in academic biobank. Forest plots show the ORs for cancers with >100 cases of genetically determined European ancestry (breast, bladder, colorectal, lung, melanoma, renal, prostate, and thyroid cancers) and African ancestry (breast, colorectal, lung, renal, prostate, and thyroid cancers). ORs are displayed as associations per SD of PRS. Breast-313, Prostate-AFR and Prostate-EUR use SNVs from previously validated PRS.^21,22^ AFR, African genetic ancestry; CRC, colorectal cancer; EUR, European genetic ancestry; OR, odds ratio; PRS, polygenic risk scores; SNV, single-nucleotide variation.

From publicly available GWAS data, we explored the association of cancer PRS on the basis of genome-wide significant SNVs with the burden of their corresponding cancer phenotype. We focused our primary analysis on cancers with >100 cases. Among individuals of European genetic ancestry, the PRS for breast (OR = 1.30, 95% CI = 1.13-1.50, *P* = 3.4 × 10^−4^), colorectal (OR = 1.26, 95% CI = 1.07-1.48, *P* = .007), melanoma (OR = 1.39, 95% CI = 1.22-1.57, *P* = 5.4 × 10^−7^), and prostate cancer (OR = 1.48, 95% CI 1.33-1.64, *P* = 1.3 ×10^−13^) were significantly associated with their respective cancer phenotypes (Figure 1, Supplemental Table 4). In addition, previously published PRSs for breast^21^ and prostate cancers^22^ were significantly associated with their respective cancers (Figure 1, Supplemental Table 4). For the remaining cancers we could not detect a significant association between the PRS and their respective cancers (Figure 1, Supplemental Table 4). Individuals of European genetic ancestry in the top PRS quintile also had a 20% greater odds of colon (OR = 1.21, 95% CI = 1.02-1.45); nearly 30% greater odds of bladder (OR = 1.28, 95% CI = 1.07-1.55) and breast (OR = 1.30, 95% CI = 1.11-1.53) cancers; and nearly 40% and 50% greater odds of melanoma (OR = 1.37, 95% CI = 1.17-1.59) and prostate cancers (OR = 1.45, 95% CI = 1.29=1.63), respectively, than those in the lowest PRS quintile (Supplemental Figure 3).

Among the cancers with >100 cases of African genetic ancestry, the PRS for thyroid (OR = 1.21, 95% CI = 1.01-1.47, *P* = .04), colon (OR = 1.29, 95% CI = 1.08-1.53, *P* = .005), and prostate (OR = 1.40, 95% CI = 1.24-1.58, *P* = 3.3 × 10^−8^) were significantly associated with their respective cancer phenotypes (Figure 1, Supplemental Table 5), and the PRS for breast cancer was approaching significance (OR = 1.12, 95% CI = 0.99-1.26, *P* = .06). For individuals of European genetic ancestry, previously published PRSs for breast^21^ and prostate cancers^22^ were significantly associated with their respective cancers in individuals of African genetic ancestry (Figure 1, Supplemental Table 5). Individuals of African genetic ancestry in the top PRS quintile also had nearly 30% greater odds of thyroid (OR = 1.30, 95% CI = 1.03-1.65), 28% greater odds of colorectal (OR = 1.28, 95% CI = 1.05-1.55), and 40% greater odds of prostate cancer (OR = 1.40, 95% CI = 1.23-1.60) than the individuals in the lowest PRS quintile (Supplemental Figure 3). The associations between PRSs and the remaining cancers are in Supplemental Table 5.

We examined the AUCs of a full logistic regression model incorporating age, sex, ancestry-specific PCs, and PRS for specific cancers (Table 2). For cancers where the PRS was significantly associated with their phenotypes (breast, colorectal, melanoma, and prostate in individuals of European genetic ancestry), the AUC for the full model ranged from 0.646 to 0.733 (Table 2). Overall, 90% to 97% of the AUC for the full model was explained by age alone (AUC for age alone: 0. 580-0.709), which increased to 96% to 99% with the inclusion of additional covariates, PCs, and sex. The inclusion of the PRS improved the discriminatory accuracy by 1% to 4%. The published breast and prostate PRS had higher AUC values, and therefore, these PRSs improved the discriminatory accuracy more than their counterparts. The inclusion of the PRS in the model was significant for breast cancer, prostate cancer, and melanomas in European genetic ancestry individuals (Table 2).

For cancers where the PRS was significantly associated with their phenotypes (breast, colorectal, prostate and thyroid) in individuals of African genetic ancestry, the AUC for the full model ranged from 0.729 to 0.817 (Table 2). Among individuals of African genetic ancestry, age explained 88% to 99% of the AUC for the full model, which increased to 99% for the full model with the inclusion of PCs and sex. The inclusion of PRS improved the discriminatory accuracy in individuals of African ancestry by <1%. In individuals of European genetic ancestry, the published breast and prostate PRS had higher AUC values, and therefore, these PRSs improved the discriminatory accuracy more than their counterparts in individuals of African genetic ancestry. However, the inclusion of the PRS in the model was significant only for the published breast and prostate PRSs in individuals of African genetic ancestry (Table 2). Additional AUC data with their respective 95% CI for the remaining cancers with <100 individuals are in Supplemental Table 6.

## Discussion

In this large retrospective case-control study, we evaluated the performance of PRSs calculated using GWAS-identified cancer risk variants for 15 major cancers in a hospital-based biobank from a large academic medical center. Among the individuals of European genetic ancestry, the PRSs for breast, colorectal, melanoma, and prostate cancers were significantly associated with their respective cancer phenotypes. Among the individuals of African genetic ancestry, the PRSs for breast, colorectal, prostate, and thyroid cancers were significantly associated with their cancer phenotype. For both individuals of European genetic ancestry and individuals of African genetic ancestry, age contributed to the highest proportion of the AUC (90%-97% among individuals of European genetic ancestry and 88%–99% among individuals of African genetic ancestry), whereas the contribution of PRSs to the existing model was higher in individuals of European genetic ancestry than in individuals of African genetic ancestry. Restricting to the cancers with >100 cases, the average AUC difference was 0.018 in individuals of European genetic ancestry compared with 0.008 in individuals of African genetic ancestry.

Although the discriminatory ability of PRS to identify patients with cancer versus controls was moderate when measured using AUCs, this was consistent with past literature in the UK Biobank.^24^ The PRS could further identify the patients of European genetic ancestry who were at higher risk of developing certain cancers. For example for breast cancer, the further discriminatory ability of PRSs in our prediction model was similar to the baseline breast cancer prediction models with addition of established clinical risk factors such as mammographic breast density,^13^ BMI,^25^ and family history.^11^

In contrast, the discriminatory ability of PRS in individuals of African genetic ancestry were extremely small for nearly all cancers tested in our study, consistent with attenuated performance of PRS in non-White patients with breast cancer.^26^ It is notable that use of well-validated SNVs^21,22^ and ancestry-specific weights^22^ led to improved performance of PRSs in individuals of African genetic ancestry. A previous meta-analysis on all PRS studies from 2008-2017, which included phenotypes such as body mass index, schizophrenia, and high-density lipoprotein, also showed that among the individuals of African genetic ancestry, the median effect size of PRSs was only 42% of that among the individuals of European genetic ancestry.^15^ It is well known that three-quarters of all GWAS have been performed exclusively in individuals of European genetic ancestry.^14^ The lack of variant representation, loci diversity, and LD patterns between populations among individuals of African genetic ancestry likely explains the smaller improvement of the model prediction compared with the genetic PCs alone. We predict that as GWAS for cancers in populations of African genetic ancestry approach the size of those currently reported in populations of European genetic ancestry, it will be possible to construct African ancestry-specific PRS with a discriminative capability similar to what we currently see in populations of European ancestry. Multiethnic PRSs have shown promise in patients of African genetic ancestry with prostate cancer.^27^

We acknowledge limitations of our analysis. First, our cancer-free group was obtained from an academic biobank at a tertiary care academic center, where patients may have higher comorbidities and not necessarily reflect the general population. Second, currently, our full model contains age, sex, PCs, and PRSs, but the precise predictive capability of PRSs in combination with other previously validated clinical attributes, such as family history, BMI, and breast density, is unknown. Follow-up of this study population with additional clinical factors could improve the performance of each cancer PRS. Furthermore, given that most were prevalent in the PMBB, we did not have enough sample size in our data set to perform separate sensitivity analyses on only incident cases. In addition, we created our PRSs from SNPs with a GWAS *P*-value significance threshold of 10^−6^. Alternative methods for creating PRS were not studied herein, including using pruning and thresholds below genome-wide significance as well as using LDpred, a Bayesian approach that takes into account the LD among various SNVs, which can improve the performance of PRS beyond pruning and thresholding.^28^ However, these methods require full summary statistics, which are not publicly available for many of the cancers. In addition, recent cancer risk models have incorporated genetic data into a polygenic hazard score^29,30^ to associate with age at cancer onset and death. Notably, polygenic hazard score performance was again better in individuals of European genetic ancestry, highlighting the need for multiancestry analyses to further identify African ancestry specific genetic loci to incorporate into genetic risk prediction. Genetic diversity needs to be prioritized in future studies to avoid systematically underperforming prediction in non-White populations.^16^

We showed that genetic liability for cancer is associated with its corresponding cancer phenotypes as classified by the EHR, with a more robust predictive capability of PRSs in individuals of European genetic ancestry than in individuals of African genetic ancestry. Expansion of GWAS in non-White populations is critically important to improve estimates of genetic risk, further tailoring PRSs as a reliable future tool for clinical cancer risk prediction across ancestry groups.

## Supplementary Material

Supplemental File

Supplemental Tables

## Figures and Tables

**Table 1:** Number of cancer cases and controls for each cancer PRS study

	European				African			
			Males				Males	
Cancer Type	Controls	Cases	*n*	%	Controls	Cases	*n*	%
Male and Female cancer cases								
Bladder	6383	176	159	90.3	5701	88	57	64.8
Colorectal	6383	147	101	68.7	5701	129	48	37.2
Esophageal	6383	41	35	85.4	5701	18	14	77.8
Glioma	6383	29	20	69.0	5701	14	8	57.1
Lung	6383	285	167	58.6	5701	187	70	37.4
Melanoma	6383	226	165	73.0	5701	15	8	53.3
Oral cavity and pharynx	6383	78	58	74.4	5701	28	21	75.0
Pancreatic	6383	46	34	73.9	5701	46	31	67.4
Renal	6383	181	148	81.8	5701	165	113	68.5
Thyroid	6383	121	65	53.7	5701	110	19	17.3
Female-only cancer cases								
Breast	2258	204			3727	303		
Endometrial	2258	47			3727	73		
Ovarian	2258	36			3727	37		
Male-only cancer cases								
Prostate	4125	457			1974	421		
Testicular	4125	18			1974	6		

African indicates African genetic ancestry, whereas European indicates European genetic ancestry.

**Table 2:** Discriminatory accuracy of components of the PRS in individuals of genetically determined African ancestry and European ancestry in the PMBB for cancers with >100 cases

	AUCs for Individuals of European Genetic Ancestry					AUC for Individuals of African Genetic Ancestry				
Cancer PRS^a^	PRS (95% CI)	Age (95% CI)	PCs, Age, Sex (95% CI)	Full Model (95% CI)	Change in AUC with Inclusion of PRS^b^ (*P*-Value)^c^	PRS (95% CI)	Age (95% CI)	PCs, Age, Sex (95% CI)	Full Model (95% CI)	Change in AUC with Inclusion of PRS^b^ (*P*-Value)^c^
Breast	0.564 (0.523-0.604)	0.580 (0.544-0.616)	0.621 (0.584-0.659)	0.646 (0.608-0.683)	0.025 (0.0303)	0.532 (0.498-0.567)	0.736 (0.712-0.760)	0.740 (0.716-0.763)	0.742 (0.717-0.766)	0.002 (0.365)
Breast-313	0.599 (0.557-0.640)	–	–	0.661 (0.623-0.698)	0.040 (0.0069)	0.593 (0.5580.627)	–	–	0.755 (0.732-0.779)	0.015 (0.017)
Prostate	0.602 (0.575-0.629)	0.709 (0.687-0.729)	0.710 (0.689-0.731)	0.733 (0.712-0.754)	0.0231 (4.52e-4)	0.569 (0.538-0.598)	0.806 (0.786-0.825)	0.810 (0.790-0.828)	0.817 (0.798-0.836)	0.008 (0.052)
Prostate-AFR	0.628 (0.602-0.654)	–	–	0.749 (0.729-0.769)	0.039 (3.43e-7)	0.624 (0.595-0.652)	–	–	0.826 (0.807-0.844)	0.016 (1.2e-3)
Prostate-EUR	0.633 (0.606-0.659)	–	–	0.752 (0.731-0.772)	0.042 (6.73e-8)	0.623 (0.594-0.652)	–	–	0.826 (0.807-0.844)	0.016 (1.6e-3)
CRC	0.568 (0.521-0.613)	0.652 (0.612-0.690)	0.679 (0.640-0.717)	0.688 (0.646-0.727)	0.009 (0.2902)	0.579 (0.530-0.625)	0.734 (0.698-0.770)	0.747 (0.712-0.782)	0.757 (0.721-0.791)	0.010 (0.123)
Thyroid	0.553 (0.501-0.606)	0.527 (0.476-0.581)	0.629 (0.579-0.675)	0.632 (0.579-0.675)	0.003 (0.7118)	0.547 (0.496-0.600)	0.638 (0.591-0.684)	0.723 (0.683-0.764)	0.729 (0.688-0.768)	0.006 (0.346)
Lung	0.525 (0.490-0.559)	0.626 (0.599-0.654)	0.647 (0.618-0.676)	0.648 (0.620-0.676)	0.001 (0.6130)	0.526 (0.483-0.568)	0.828 (0.805-0.851)	0.837 (0.814-0.858)	0.837 (0.815-0.859)	0.000 (0.615)
Renal	0.516 (0.476-0.557)	0.620 (0.583-0.655)	0.683 (0.643-0.720)	0.683 (0.643-0.721)	0.000 (0.9725)	0.532 (0.485-0.576)	0.76 (0.727-0.790)	0.797 (0.766-0.826)	0.799 (0.767-0.828)	0.002 (0.422)
Melanoma	0.612 (0.576-0.647)	0.654 (0.621-0.686)	0.692 (0.654-0.728)	0.708 (0.672-0.744)	0.016 (0.0213)	–	–	–	–	–
Bladder	0.531 (0.489-0.574)	0.702 (0.700-0.733)	0.77 (0.737-0.802)	0.772 (0.740-0.802)	0.002 (0.5268)	–	–	–	–	–

*AFR*, African genetic ancestry; *AUC*, area under the curve; *CRC*, colorectal cancer; *EUR*, European genetic ancestry; *GWAS*, Genome-wide association studies; *PC*, principal components; *PMBB*, Penn Medicine BioBank; *PRS*, polygenic risk score; *SNV*, single-nucleotide variation.

a PRS derived from all reported GWAS SNVs with *P* value < 10^−6^ unless indicated; Breast-313 has published SNVs from Kramer et al,^21^ Prostate-AFR and Prostate-EUR has published SNVs and genetic ancestry stratified weights from Conti et al.^22^

b AUC with inclusion of PRS = AUC of full model – AUC for model of PCs, Age, Sex.

c Bonferroni corrected *P* value threshold 1.7e-03.

## Data Availability

Summary statistics used to calculate PRS for each of the 15 cancers are available from the GWAS catalog, as detailed in Supplemental Table 2. Individual-level data for the Penn Medicine BiobBank are not publicly available because of research participant privacy concerns; however, requests from accredited researchers for access to individual-level data relevant to this manuscript can be made by contacting the corresponding author.

## Members of Regeneron Genetics Center

Goncalo Abecasis, Xiaodong Bai, Suganthi Balasubramanian, Aris Baras, Andrew Blumenfeld, Boris Boutkov, Michael Cantor, Giovanni Coppola, Aris Economides, Gisu Eom, Lukas Habegger, Alicia Hawes, Marcus B. Jones, Shareef Khalid, Olga Krasheninina, Rouel Lanche, Luca A. Lotta, Adam J. Mansfield, Evan K. Maxwell, Jason Mighty, Lyndon J. Mitnaul, Mrunali Nafde, Sean O’Keeffe, Max Orelus, John D. Overton, Razvan Panea, Tommy Polanco, Ayesha Rasool, Jeffrey G. Reid, William Salerno, Jeffrey C. Staples, Alan Shuldiner, Christina Beechert, Caitlin Forsythe, Erin D. Fuller, Zhenhua Gu, Michael Lattari, Alexander Lopez, Kia Manoochehri, John D. Overton, Manasi Pradhan, Thomas D. Schleicher, Maria Sotiropoulos Padilla, Ricardo H. Ulloa, Louis Widom, Sarah E. Wolf.
