# Supplementary material for: Performance of polygenic risk scores for cancer prediction in a racially diverse academic biobank
Source: Genet Med. Author manuscript; Available in PMC 2022 Nov 22. (PMC9680700; doi:10.1016/j.gim.2021.10.015)
Supplement: Supplemental File [file NIHMS1848614-supplement-Supplemental_File.pdf]

PPV of ICD9/10 codes for prostate cancer

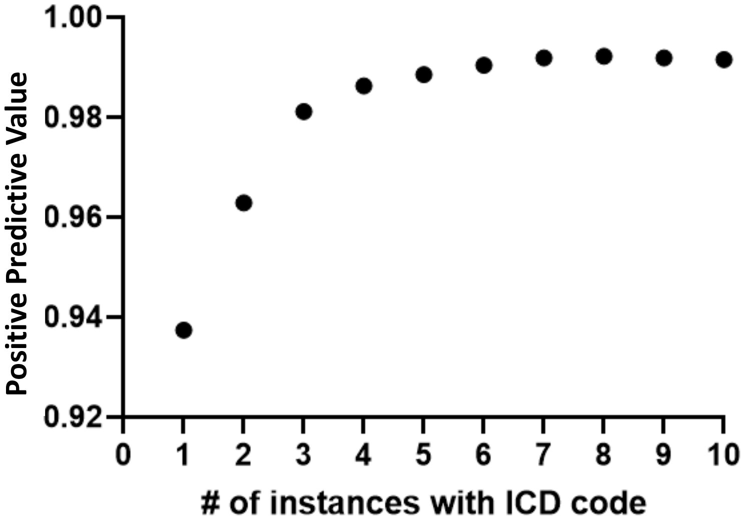

# of ICD codes	1	2	3	4	5	6	7	8	9	10
Total	2365	2129	1967	1897	1833	1775	1735	1681	1604	1540
True positives	2217	2050	1930	1871	1812	1758	1721	1668	1591	1527
PPV	0.937	0.963	0.981	0.986	0.989	0.990	0.992	0.992	0.992	0.992

Supplementary Figure 1: Positive predictive value of ICD9/10 billing code based identification of cancer cases in PMBB. Prostate cancer cases with the indicated number of instances of ICD9/10 codes for prostate cancer (C61 parent) and those in the cancer registry were manually reviewed to determine if truly had prostate cancer.

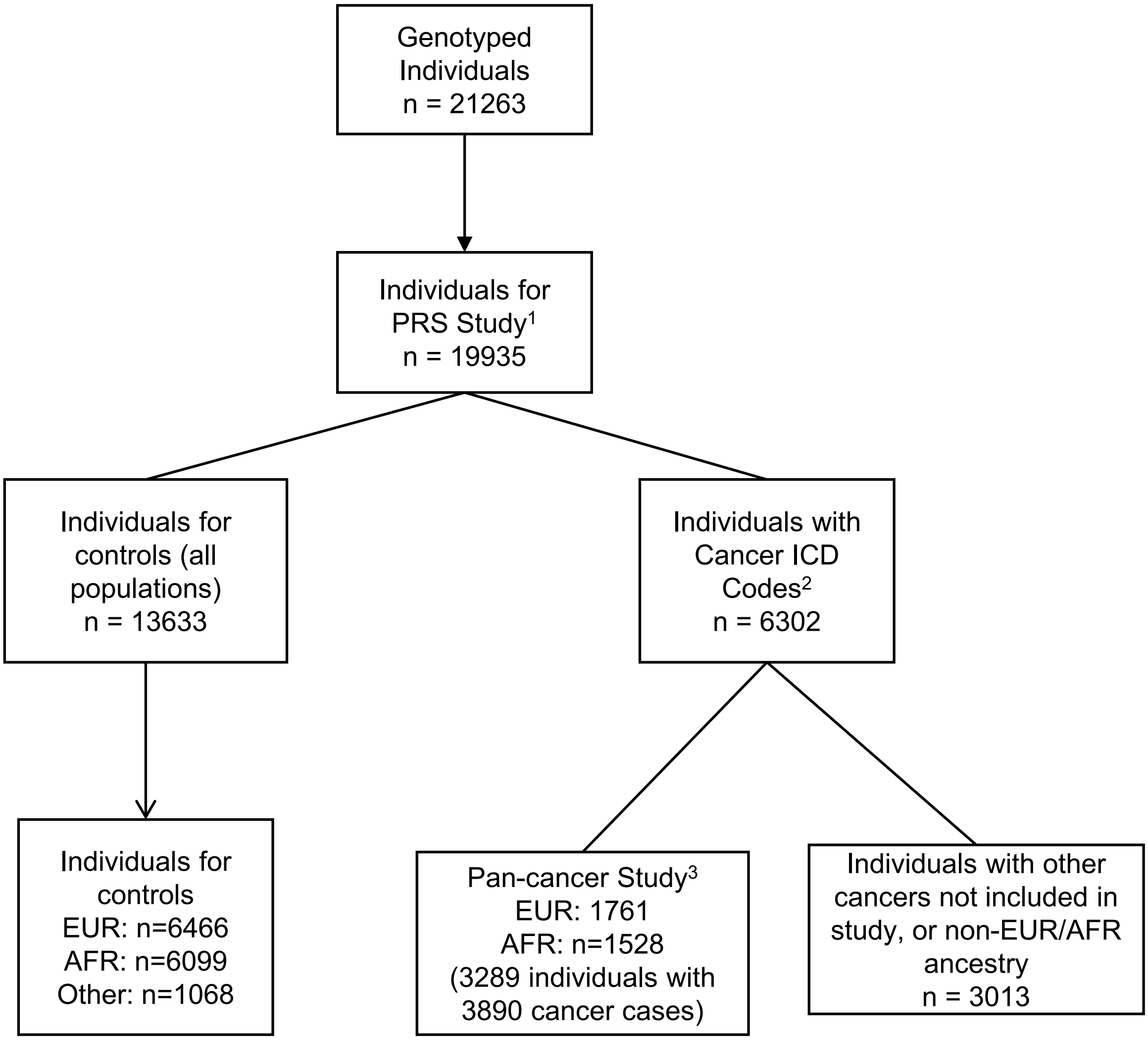

Supplementary Figure 2: Cohort identification among genotyped individuals in PMBB. From 21,263 individuals with SNP genotyping, 19,935 passed quality control measures. The number of instances of any ICD9/10 billing codes in the Electronic Health Record were obtained for each individual in the PMBB. Eigenstrat Principal Components Analysis (PCA) was used to identify the genetic substructure of the entire PMBB population and to assign genetically derived African ancestry (AFR), European ancestry (EUR), or Other ancestry to each individual. Cancer-free individuals had no ICD9/10 codes for any invasive cancer, nor any codes for benign, in situ or secondary neoplasms. Individuals with one or more cancers had at least one instance in the electronic health record of an ICD9/10 code corresponding to one or more of15 cancers with GWAS study summary statistics. Individuals with other cancers were excluded from the study.

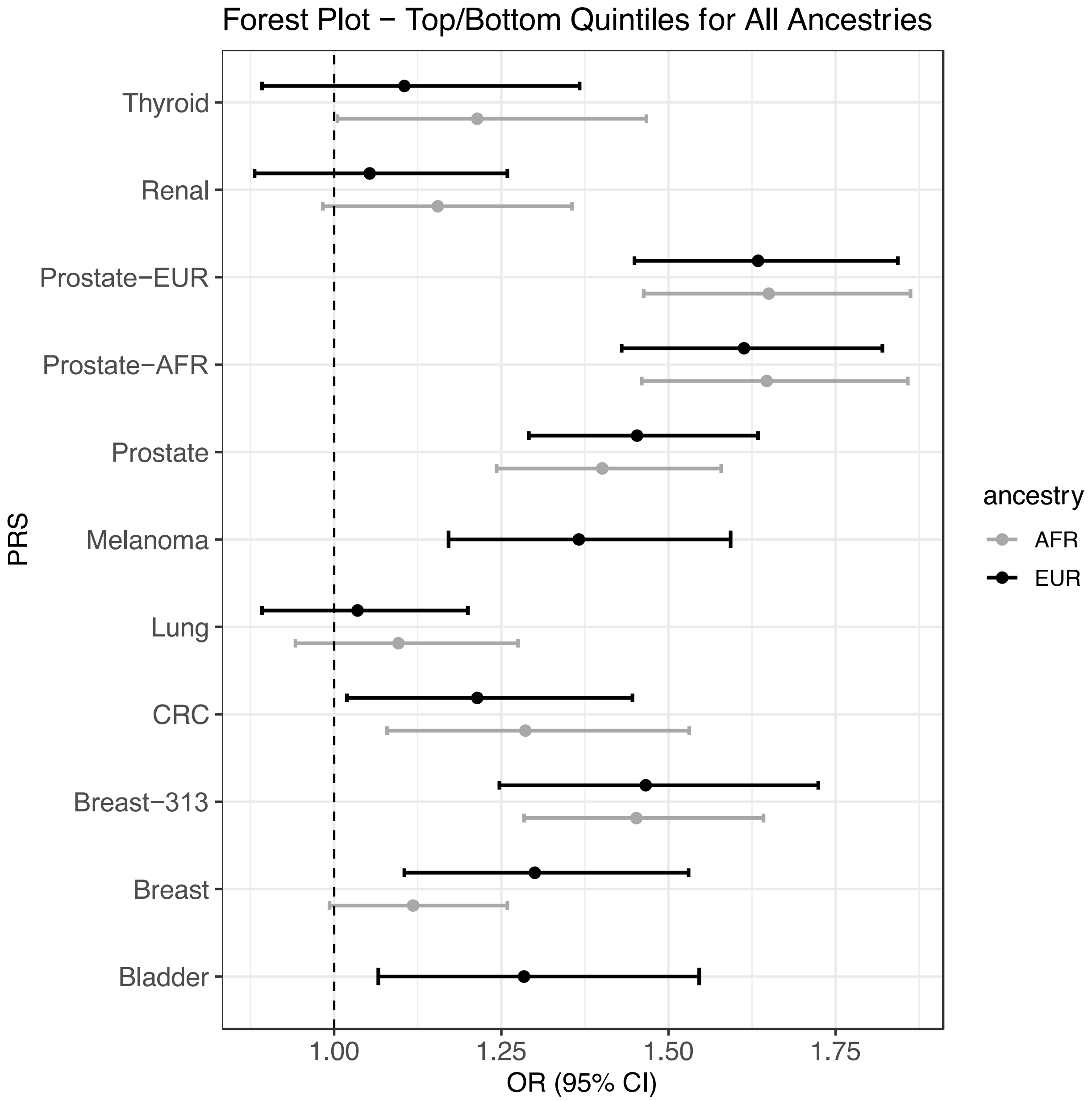

Supplementary Figure 3: Association of PRS with cancer in AFR and EUR academic biobank participants. Forest plots demonstrating the odds ratio for specific cancers of a PRS comparing the top to bottom quintile of scores for cancers with greater than 100 cases in genetically determined EUR (European; breast, bladder, colorectal, lung, melanoma, renal, prostate and thyroid) and AFR (African; breast, colorectal, lung, renal, prostate and thyroid) individuals. Breast-313, Prostate- AFR and Prostate-EUR use SNPs from previously validated PRS[21,22]
